# A Multifactorial Presentation of Infection and Thrombosis: Temporal Clustering of Pneumonia, Hepatic Abscess, Bilateral Deep Vein Thromboses (DVTs), and Appendicitis in a Child

**DOI:** 10.7759/cureus.102274

**Published:** 2026-01-25

**Authors:** Leonardo Bonifanti, Brooke Heyer, Vivian Calderon, Jossias Genao-Cruz, Judith Cornely

**Affiliations:** 1 College of Osteopathic Medicine, Nova Southeastern University Dr. Kiran C. Patel College of Osteopathic Medicine, Fort Lauderdale, USA; 2 Pediatrics, Broward Health Medical Center, Fort Lauderdale, USA

**Keywords:** beta thalassemia trait, blood hypercoagulability, deep vein thrombosis (dvt), multisystem disease, paediatric empyema, pediatric infection, pyogenic hepatic abscess, ruptured appendicitis, sickle cell trait

## Abstract

Multisystem infectious and thrombotic presentations are rare in pediatric patients and often raise concern for an underlying immunologic or hematologic disorder. We report a case of a 13-year-old male with sickle cell trait and beta-thalassemia trait heterozygote who developed extensive bilateral deep vein thromboses (DVTs), a large hepatic abscess, right-sided pneumonia with empyema, and subsequently perforated appendicitis with intra-abdominal abscesses. The patient required broad-spectrum antimicrobial therapy, anticoagulation, percutaneous abscess drainage, and emergent surgical intervention. An extensive evaluation excluded inherited thrombophilias, autoimmune disease, and chronic granulomatous disease. The close temporal clustering of these events highlights a multifactorial process involving systemic inflammation, infection, and acquired hypercoagulability. This case underscores the importance of multidisciplinary management and longitudinal follow-up in pediatric patients presenting with recurrent or multifocal infections and thrombosis.

## Introduction

Multisystem involvement in pediatric patients presenting with both thrombotic and infectious complications is uncommon and often raises suspicion for an underlying immunologic, hematologic, or systemic inflammatory disorder [1]. While hepatic abscesses, deep vein thromboses (DVTs), and appendicitis are each individually well-characterized in children, they differ markedly in baseline incidence and typical predisposing factors, and rarely occur in temporal association, making their sequential or concurrent occurrence in a pediatric population uncommon and diagnostically challenging. Pediatric DVTs, in particular, are infrequent and most often linked to identifiable provoking factors such as central venous catheters, trauma, or congenital thrombophilias [2]. Similarly, hepatic abscesses in immunocompetent children are uncommon and usually associated with hematogenous spread, specific pathogens, or structural abnormalities rather than routine intra-abdominal pathology [3]. Appendicitis, although common in pediatrics, typically presents as an isolated intra-abdominal process, with systemic infectious or thrombotic complications occurring only rarely, most often in the setting of perforation or delayed diagnosis. Taken together, the distinct epidemiology, pathophysiology, and complication profiles of these conditions underscore the rarity of their temporal clustering and highlight the diagnostic complexity when they present in combination.

This case describes a 13-year-old Black male who presented with bilateral DVTs, a large hepatic abscess with polymicrobial growth, and right lower lobe pneumonia with an empyema, and was later found to have perforated appendicitis with multiple intra-abdominal abscesses. This patient had multiple barriers to access to care, including a difficult social situation, low socioeconomic status, and an inability to advocate for himself. This led to a lack of prior medical workup. Despite extensive evaluation, including infectious, hematologic, and immunologic workups, a single unifying etiology has yet to be identified. The constellation and timing of these findings suggest a complex underlying pathophysiology that bridges infection, coagulation, and immune regulation.

We present this case to highlight the importance of maintaining a broad differential diagnosis in pediatric patients with recurrent or multifocal infections and thrombotic events, and to emphasize the diagnostic value of multidisciplinary collaboration when systemic disease is suspected yet not fully defined.

## Case presentation

A 13-year-old Black male with unknown past medical history presented to the emergency department (ED) with a several-month history of progressive, unintentional weight loss, intermittent non-bloody, non-bilious emesis, decreased oral intake, generalized weakness, and self-reported fevers and chills. It is presumed that he did not receive a prior work-up due to numerous social and financial barriers to care access. Five days prior to admission, he developed left lower extremity swelling and bilateral leg pain, which had progressively worsened.

On arrival, he appeared cachectic, was unable to ambulate, and had bilateral lower extremity edema. Initial laboratory evaluation revealed leukocytosis (24.2 × 10³/μL), severe anemia (hemoglobin 5.8 g/dL), thrombocytosis (platelets 538 × 10³/μL), elevated inflammatory markers (erythrocyte sedimentation rate (ESR) 75 mm/hr, C-reactive protein (CRP) 20.4 mg/dL), elevated lactate (2.5 mmol/L), and hypoalbuminemia (3.0 g/dL). Coagulation studies showed a mildly prolonged prothrombin time (PT 16.4 sec, international normalized ratio (INR) 1.3), and urinalysis revealed mild proteinuria with hyaline casts and rare bacteria. His hemoglobin electrophoresis suggested a compound heterozygote sickle cell disease of sickle cell-beta thalassemia trait. No proprietary clinical scoring systems, questionnaires, or licensed diagnostic tools were used in this study; all laboratory tests and diagnostic criteria applied are part of standard clinical care and derived from publicly available international consensus guidelines. Laboratory abnormalities were interpreted using established pediatric reference ranges and international consensus guidelines for pediatric venous thromboembolism and antiphospholipid antibody testing and are summarized in Table 1 [4-8].

**Table 1:** Peak derangements in hematologic and inflammatory laboratory findings WBC, white blood cell count; MCV, mean corpuscular volume; RDW, red cell distribution width; ESR, erythrocyte sedimentation rate; CRP, C-reactive protein

Parameter	Admission (5/26/25)	Peak abnormality	Pre-discharge / improving	Reference range
WBC (×10³/µL)	24.2	24.2	13.9	4.5–13.5
Hemoglobin (g/dL)	5.8	5.8	10.0	11.5–15.5
Platelets (×10³/µL)	538	538	499	150–450
MCV (fL)	Low (microcytic)	74.5	77.9	77–95
RDW (%)	22.7	22.7	21.1	11.5–14.5
ESR (mm/hr)	75	75	35	0–20
CRP (mg/dL)	20.4	31.97	8.59	<1.0

In the setting of bilateral lower extremity DVTs, a comprehensive coagulation and thrombophilia evaluation was performed. Testing demonstrated reduced antithrombin III levels and a transiently positive lupus anticoagulant that normalized on repeat assessment, while protein C, protein S, Factor V Leiden, prothrombin gene mutation, and antiphospholipid antibodies were negative. These findings were interpreted as consistent with an acquired, inflammation-associated hypercoagulable state rather than an inherited thrombophilia and are summarized in Table 2 [6-9].

**Table 2:** Coagulation and thrombophilia evaluation PT, prothrombin time; INR, international normalized ratio; aPTT, activated partial thromboplastin time

Test	Result	Reference range	Interpretation
PT (sec)	16.4	11–14	Mild prolongation
INR	1.3	0.8–1.2	Mildly elevated
aPTT (sec)	29.9	25–35	Normal
Antithrombin III (%)	58	80–120	Decreased
Protein C antigen	Normal	70–140%	Normal
Protein S antigen	66	65–140%	Low-normal
Lupus anticoagulant (initial)	Positive	Negative	Likely transient
Lupus anticoagulant (repeat)	Negative	Negative	Normalized
Factor V Leiden	Negative	Negative	Excluded
Prothrombin gene mutation	Negative	Negative	Excluded
β2-glycoprotein I Abs	Negative	Negative	Excluded
Anticardiolipin Abs	Negative	Negative	Excluded

**Figure 1:**
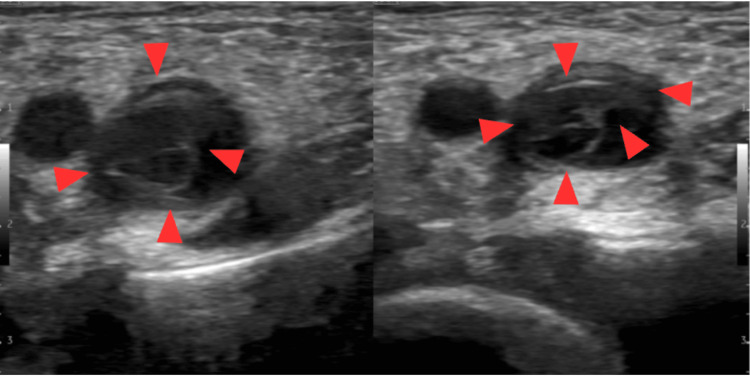
Transverse (short-axis) ultrasound image of the femoral vein demonstrating intraluminal thrombus (red arrows) extending from the common femoral to the popliteal vein.

**Figure 2:**
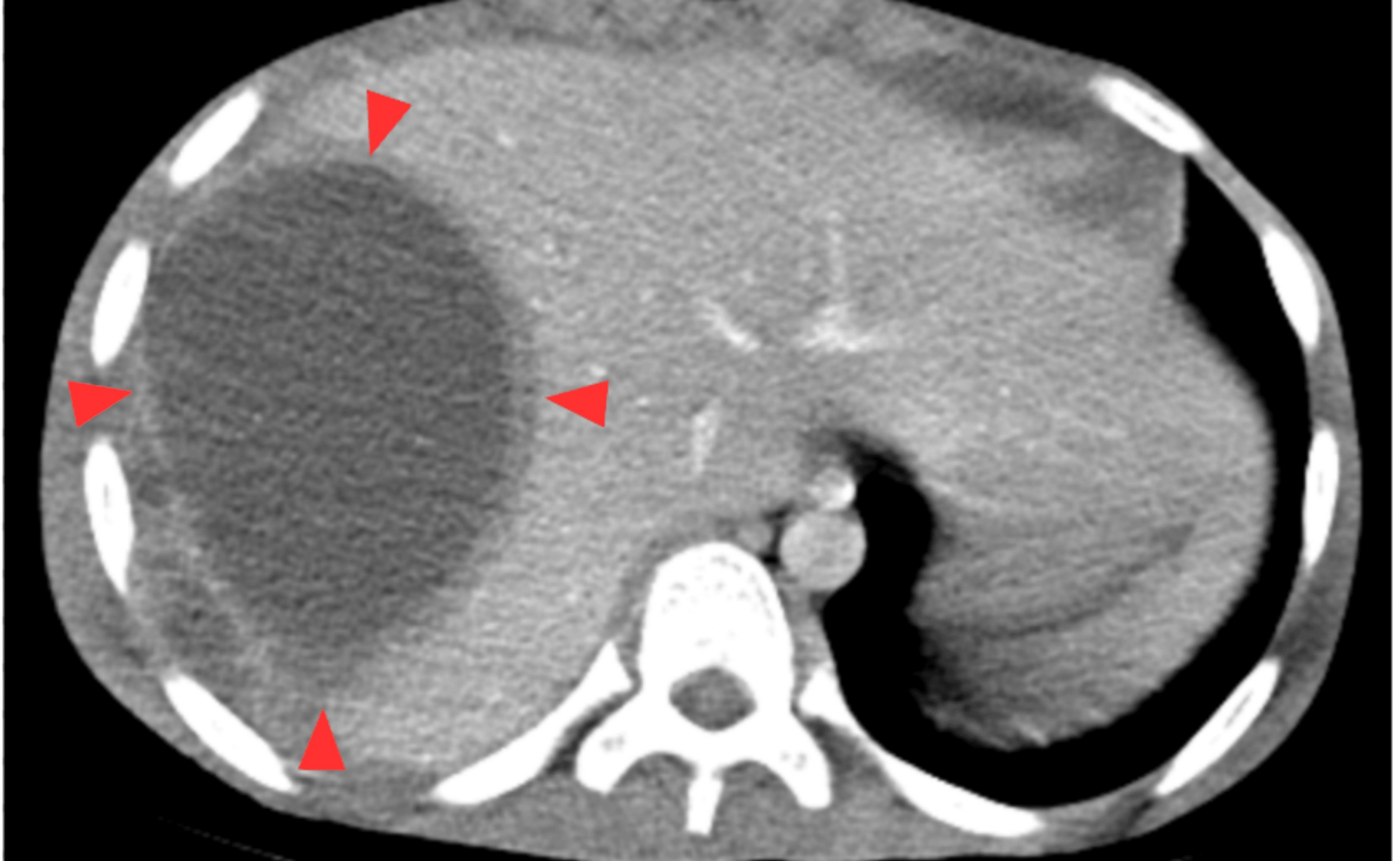
Non-contrast transverse plane CT showing liver abscess measuring 10.1 cm by 9.0 cm by 12.5 cm in the right lobe of the liver with an enhancing wall measuring 6 mm.

**Figure 3:**
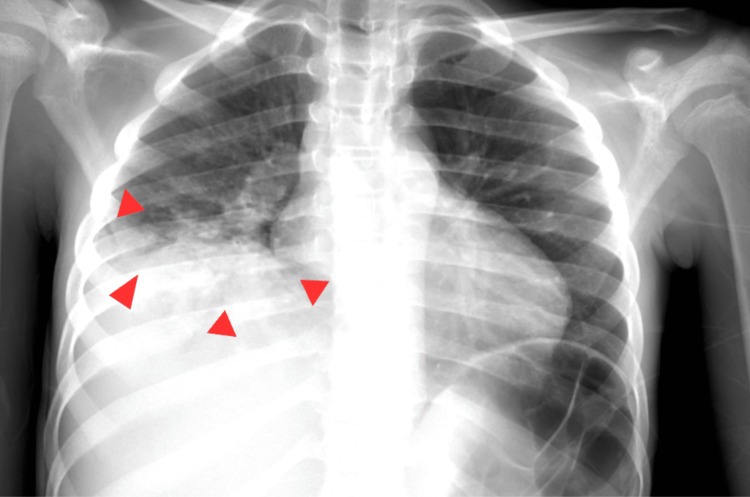
AP chest radiograph demonstrates a right-sided loculated pleural collection with associated pleural thickening and adjacent compressive atelectasis, consistent with empyema

Transverse (short-axis) ultrasound image of the femoral vein demonstrated extensive bilateral lower-extremity DVTs (Figure 1), a large hepatic abscess (Figure 2), and right middle and lower lobe pneumonia with a small associated empyema (Figure 3).

In the ED, the patient became hypoxic and required 2 L of oxygen via nasal cannula. Initial management included intravenous cefepime, vancomycin, morphine, ibuprofen, and fluid resuscitation. Broad-spectrum coverage was escalated to meropenem following imaging suggestive of hepatic abscess. He was admitted to the pediatric acute care unit in stable condition and transitioned to clindamycin, which he completed as a 21-day course. In addition, enoxaparin was initiated for anticoagulation upon transfer to the pediatric acute care unit, and Anti-Xa (Anti-factor 10a) levels were monitored.

On hospital day 3, gastrointestinal polymerase chain reaction (PCR) testing returned positive for rotavirus. Fecal calprotectin and fecal pancreatic elastase were obtained to further evaluate gastrointestinal involvement, as these studies provide objective evidence of intestinal inflammation and digestive dysfunction not captured by systemic laboratory markers alone. On day 4, interventional radiology-guided hepatic abscess drainage with indwelling drain placement was performed. Cultures revealed heavy growth of Bacteroides ovatus and light growth of Streptococcus constellatus subsp. pharyngis. Furthermore, a comprehensive autoimmune and hypercoagulability evaluation was pursued. Initial testing showed a transiently positive lupus anticoagulant with prolonged Activated Partial Thromboplastin Time-Lupus Anticoagulant (PTT-LA) and Dilute Russell's Viper Venom Time (dRVVT) screens, though repeat testing normalized, supporting a likely inflammation-related false positive rather than true antiphospholipid syndrome. Additional thrombophilia studies, including Protein S antigen, Factor V Leiden mutation, prothrombin gene mutation, Protein C antigen, and β2-glycoprotein antibodies, were all within reference ranges or negative. Antinuclear Antibody (ANA) and broader autoimmune serologies were also negative. Normal neutrophil oxidative burst ruled out chronic granulomatous disease (CGD). The overall workup demonstrated no persistent autoimmune etiology and no inherited thrombophilia, suggesting that the patient’s thrombotic events were more plausibly driven by acute infection-related hypercoagulability rather than a chronic prothrombotic disorder. The liver drain was removed after adequate output and clinical improvement, and the patient was transitioned to oral rivaroxaban on day 18. He was discharged on 6/23 (day 19) with rivaroxaban and ferrous sulfate for iron-deficiency anemia, with instructions to follow up with hematology and oncology for continuous monitoring and medication optimization. 

At the time of discharge from his index hospitalization on 6/23/2025 (hospital day 19), the patient had improving but unresolved laboratory abnormalities, including residual leukocytosis (WBC 13.9 ×10³/µL), persistent microcytic anemia (hemoglobin 10.0 g/dL, MCV 77.9 fL, RDW 22.7%), thrombocytosis (platelets 499 ×10³/µL), hypoalbuminemia (2.9 g/dL), and an elevated CRP of 8.59 mg/dL following a peak of 31.97 mg/dL. Twelve days later, he returned to the emergency department with acute abdominal pain, vomiting, leukocytosis (WBC 18.77 ×10³/µL with 81.4% neutrophils; absolute neutrophil count 15.29 ×10³/µL), and peritoneal signs. He was taken emergently to the operating room, where he was found to have perforated appendicitis with purulent peritonitis and multiple interloop pelvic abscesses, and underwent operative management. Postoperatively, he developed persistent fevers, tachycardia, hypotension, and an acute inflammatory surge, with CRP rising to 31.97 mg/dL and remaining elevated at 31.45 mg/dL. He was treated with broad-spectrum intravenous antibiotics, including vancomycin, with therapeutic trough levels achieved, and his course was further complicated by an acute decline in hemoglobin to a nadir of 8.4 g/dL without reticulocytosis, persistent hypoalbuminemia (2.4 g/dL), and metabolic derangements consistent with systemic inflammatory response physiology. Serial laboratory monitoring demonstrated gradual improvement, with CRP decreasing to 20.4 mg/dL on 6/28/2025 and 8.59 mg/dL by 6/30/2025, after which he was deemed surgically cleared for discharge with plans for continued outpatient infectious and immunologic evaluation. 

## Discussion

This case describes a diagnostically challenging and clinically severe presentation in a 13-year-old Black male with sickle-cell beta thalassemia trait who developed multisystem complications, including bilateral DVTs, hepatic abscess, pneumonia with empyema, and subsequently, perforated appendicitis with intra-abdominal abscesses. The temporal clustering of these pathologies over a one-month period in the absence of significant prior medical history or known predisposing factors raises important questions about underlying systemic dysfunction and highlights the need for multidisciplinary evaluation in complex pediatric cases.

Infection induces hypercoagulability through multiple interconnected mechanisms involving inflammation, endothelial dysfunction, and disruption of normal hemostatic balance. Systemic inflammation resulting from severe infection leads to activation of coagulation due to tissue factor-mediated thrombin generation, downregulation of physiological anticoagulant mechanisms (including the protein C pathway and antithrombin), and inhibition of fibrinolysis, with proinflammatory cytokines playing a central role in these differential effects on the coagulation and fibrinolysis pathways. [10] The endothelium loses its intrinsic antithrombotic properties due to inflammatory mediators and direct pathogen effects, promoting platelet activation and creating a prothrombotic microenvironment that can range from insignificant laboratory changes to severe disseminated intravascular coagulation [10]. In pediatric populations, infection and sepsis represent significant independent risk factors for thrombosis, with a meta-analysis demonstrating an odds ratio of 1.95 (P < 0.01) in children and neonates in intensive care units, often occurring in conjunction with other risk factors such as central venous catheters and critical illness [11].

While each of the individual findings can occur in isolation, their combination is highly unusual. Pediatric hepatic abscesses are rare, with an incidence of less than 0.1 per 100,000 in developed countries, and are typically associated with immunodeficiency, hematogenous spread, or intra-abdominal infections [12]. DVTs in children are also uncommon, with an estimated incidence of 0.07 to 0.14 per 10,000, and are often linked to central venous catheters, trauma, malignancy, or congenital thrombophilia [13,14]. This patient had extensive DVTs found in both the right and left common femoral vein, saphenous vein, proximal, mid, and distal femoral vein, along with the popliteal vein. Appendicitis is common in pediatrics, but its occurrence after a series of systemic infectious and thrombotic events suggests either a coincidental progression or a shared pathophysiologic mechanism, such as altered gut barrier function or immune dysregulation.

The patient’s diagnostic workup yielded several abnormalities that may offer partial explanations. Hemoglobin electrophoresis was consistent with sickle-cell beta thalassemia trait, which contributes to microcytic anemia but does not typically predispose to infection or thrombosis [15]. His antithrombin III level was low (58%), suggesting a possible acquired or inherited thrombophilia, although protein C, protein S, and Factor V Leiden testing were unremarkable. A transiently positive lupus anticoagulant was later found to be negative on repeat, likely influenced by concurrent anticoagulation, as false-positive lupus anticoagulant results are well-documented during anticoagulant therapy [16]. Immunologic testing revealed elevated total IgG and all IgG subclasses, raising concern for immune dysregulation; however, hypergammaglobulinemia may be reactive and nonspecific in the setting of systemic inflammation or infection [17].

Fecal calprotectin was mildly elevated, and pancreatic elastase was low, raising concern for exocrine pancreatic insufficiency or an underlying gastrointestinal immune process. While the findings were nonspecific, these abnormalities support the hypothesis of altered intestinal barrier function. Hepatic abscess culture revealed Bacteroides ovatus and Streptococcus constellatus, both anaerobic or facultative anaerobic organisms of gastrointestinal origin, suggesting microbial translocation as a potential mechanism. Additionally, gastrointestinal PCR was positive for rotavirus, which has been associated with transient disruption of intestinal tight junctions and increased bacterial translocation in animal models [18,19].

The absence of a unifying diagnosis reflects the limitations of current diagnostic tools when evaluating evolving multisystem pathology in pediatric patients. Chronic granulomatous disease was ruled out by a normal dihydrorhodamine (DHR) test, and autoimmune and thrombophilia panels were largely unremarkable. However, the combination of infectious, hematologic, and gastrointestinal findings remains concerning for a possible undiagnosed primary immunodeficiency or autoinflammatory condition. In such cases, extended immunologic testing and genetic sequencing may eventually yield diagnostic clarity.

This case underscores the importance of maintaining a broad differential when systemic involvement spans multiple organ systems in pediatric patients. Early multidisciplinary collaboration, longitudinal reassessment of symptoms, and continued follow-up are crucial for both diagnostic clarity and optimal patient care in similarly complex clinical scenarios.

## Conclusions

This case illustrates a rare and diagnostically complex presentation of recurrent, multifocal infections and thrombotic events in a previously healthy pediatric patient. The sequential development of bilateral DVTs, hepatic abscess, pneumonia with empyema, and later perforated appendicitis with intra-abdominal abscesses underscores the importance of considering underlying immune or hematologic disorders when multiple organ systems are involved. Despite an extensive diagnostic workup, no unifying etiology has yet been confirmed, highlighting the real-world challenges clinicians face when evaluating systemic illness in children. Early recognition, aggressive multidisciplinary management, and continued longitudinal follow-up remain critical in optimizing outcomes and uncovering potential underlying pathology.
